# Abatacept: A Promising Repurposed Solution for Myocardial Infarction-Induced Inflammation in Rat Models

**DOI:** 10.1155/2024/3534104

**Published:** 2024-03-13

**Authors:** Vipin Kumar Verma, Ekta Mutneja, Salma Malik, Anil Kumar Sahu, Vaishali Prajapati, Priya Bhardwaj, Ruma Ray, Tapas Chandra Nag, Jagriti Bhatia, Dharamvir Singh Arya

**Affiliations:** ^1^Cardiovascular Research Laboratory, Department of Pharmacology, All India Institute of Medical Sciences, New Delhi-110029, India; ^2^Cardiac Pathology Laboratory, Department of Pathology, All India Institute of Medical Sciences, New Delhi-110029, India; ^3^Department of Anatomy, All India Institute of Medical Sciences, New Delhi-110029, India

## Abstract

Myocardial infarction (MI) is irreversible damage to the myocardial tissue caused by prolonged ischemia/hypoxia, subsequently leading to loss of contractile function and myocardial damage. However, after a perilous period, ischemia-reperfusion (IR) itself causes the generation of oxygen free radicals, disturbance in cation homeostasis, depletion of cellular energy stores, and activation of innate and adaptive immune responses. The present study employed Abatacept (ABT), which is an anti-inflammatory drug, originally used as an antirheumatic response agent. To investigate the cardioprotective potential of ABT, primarily, the dose was optimized in a chemically induced model of myocardial necrosis. Thereafter, ABT optimized the dose of 5 mg/kg s.c. OD was investigated for its cardioprotective potential in a surgical model of myocardial IR injury, where animals (*n* = 30) were randomized into five groups: Sham, IR-C, Telmi10 + IR (Telmisartan, 10 mg/kg oral OD), ABT5 + IR, ABT *perse*. ABT and telmisartan were administered for 21 days. On the 21st day, animals were subjected to LAD coronary artery occlusion for 60 min, followed by reperfusion for 45 min. Further, the cardioprotective potential was assessed through hemodynamic parameters, oxidant–antioxidant biochemical enzymatic parameters, cardiac injury, inflammatory markers, histopathological analysis, TUNEL assay, and immunohistochemical evaluation, followed by immunoblotting to explore signaling pathways. The statistics were performed by one-way analysis of variance, followed by the Tukey comparison post hoc tests. Noteworthy, 21 days of ABT pretreatment amended the hemodynamic and ventricular functions in the rat models of MI. The cardioprotective potential of ABT is accompanied by inhibiting MAP kinase signaling and modulating Nrf-2/HO-1 proteins downstream signaling cascade. Overall, the present work bolsters the previously known anti-inflammatory role of ABT in MI and contributes a mechanistic insight and application of clinically approved drugs in averting the activation of inflammatory response.

## 1. Introduction

Myocardial infarction (MI) is the most prevalent devastating coronary-associated pathology among cardiovascular diseases (CVDs) that occurs as a consequence of inadequate blood flow to the cardiomyocytes [1]. Cardiomyocytes are dependent on coronary blood supply *vis-a-vis* its metabolic requirement; however, prolonged ischemia/hypoxia or when ischemic condition exceeds a critical threshold leads to necrosis and apoptosis of cardiomyocytes and ultimately culminates into the loss of contractile function and myocardial damage [2, 3]. In addition, MI occurs when a coronary artery becomes occluded following the erosion of an atherosclerotic plaque, which triggers the recruitment and accumulation of leukocytes and neutrophils on the lesion and ultimately leads to coronary thrombosis [4]. This event can also trigger coronary vasospasm [5]. Furthermore, if the blood is restored through reperfusion to the at-risk myocardium, more heart muscle can be saved from irreversible damage [6]. Paradoxically, after a definite period, reperfusion itself causes the generation of reactive oxygen species (ROS), disturbance in cation homeostasis, and depletion of cellular energy stores, resulting in severe damage to the myocardium, a phenomenon called myocardial reperfusion injury [7]. Also, these altered etiologies in the myocardial tissue may elicit arrhythmias, contractile dysfunction, and structural damage, in addition to endothelial dysfunction and coronary vasoconstriction [8–11]. ROS also regulates the expression of several proinflammatory genes that, in turn, stimulate multiple cell death pathways [7, 9]. Also, the inflammatory changes may cause the death of cardiomyocytes and infarction expansion, resulting in activation of the innate immune system that triggers inflammatory reactions. The activated immune system orchestrates post-infarction tissue remodeling and repair [12, 13]. The regenerative and angiogenic immune reactions program to resolve inflammation and regeneration of injured myocardium. Moreover, immune cells clear necrotic cells and extracellular matrix (ECM) debris, subsequently providing a milieu required for migration, proliferation, and differentiation of fibroblasts and endothelial cells, necessary for the reconstruction of ECM, neo-vascularization and, ultimately, heart recovery [14]. Therefore, targeting inflammatory pathways and key processes in the immune system aids in alleviating postischemic dysfunction. However, despite the in-depth understanding of inflammation in the pathophysiology of coronary occlusion, the development of anti-inflammatory therapies to attenuate MI poses a major challenge [15].

Abatacept (ABT) is a potent anti-inflammatory agent well-known as a bDMARD (biological Disease-Modifying Antirheumatic Drug) for rheumatoid arthritis [16, 17]. It is a recombinant form of a fusion protein that is expressed on the extracellular area (or domain) of human cytotoxic T-lymphocytes. It inhibits the activation of T-cells by inhibiting the CD80/CD86: CD28 costimulation and thereby prevents the inflammatory response following injury [16, 18]. Several studies have demonstrated the potent anti-inflammatory potential of ABT via halting the release of inflammatory markers such as chemokines and cytokines [18–20]. To the given anti-inflammatory properties of ABT, we have evaluated its cardio-protective potential in rat models of MI. Herein, we examine the optimal dose of ABT in rats following the isoproterenol-induced model of myocardial necrosis, with a focus on its putative implications as a therapeutic in myocardial ischemia-reperfusion injury (MIRI).

## 2. Materials and Methods

Male Wistar albino rats (150–200 g) were purchased from the central animal facility of All India Institute of Medical Sciences, New Delhi, India. Animals were acclimatized in the departmental animal house in polypropylene cages of 40 × 25 × 15 cm^3^ size at 25 ± 2°C in an air-conditioned room. Relative humidity at 60% ± 5% with a 12:12 light–dark cycle was maintained, and animals were fed with a chow diet and water *ad libitum*.

### 2.1. Drugs and Chemicals

Isoproterenol hydrochloride (ISO) was obtained from Sigma-Aldrich Chemicals (India) (5984-95-2). ABT, i.e., test drug, was provided by Bristol Myers Squibb, USA as a gift sample. Primary antibodies and kits used in the experiments are listed in *Supplementary 1* and *2*, respectively. Secondary antibodies such as horseradish peroxidase (HRP) conjugated Goat anti-rabbit IgG were purchased from Thermo Fisher Scientific, USA (31460), and fluorescent (Alexafluoro488 labeled) antibody was from Abcam, California, USA (ab150113). Creatine Kinase-MB (1102070210) isoenzyme and lactate dehydrogenase (LDH) (1102160025) kits were procured and obtained from Coral Clinical Systems, India. Terminal deoxynucleotidyl transferase dUTP nick end labeling assay kit was purchased from Thermo Fisher Scientific, USA (TUNEL) (A23210). The rest of the chemicals used in the experiments were of molecular biology grade from SRL Chemicals Pvt. Ltd.

### 2.2. Study Design

**Figure 1:**
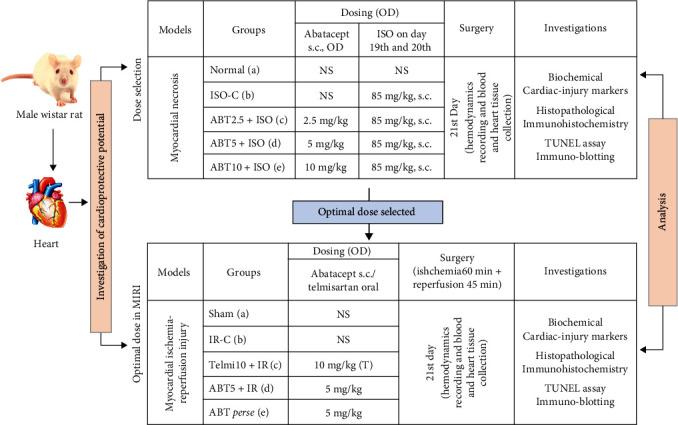
Schematic representation of study protocol. Investigation of cardioprotective potential of Abatacept was performed using myocardial necrosis model where a dose of Abatacept was optimized, and thereafter, an optimized dose of Abatacept was used in the model of myocardial infarction ischemia injury. NS, normal saline; ISO-C, isoproterenol control; ABT2.5 + ISO, Abatacept 2.5 mg/kg + isoproterenol; ABT5 + ISO, Abatacept 5 mg/kg + isoproterenol; ABT10 + ISO, Abatacept 10 mg/kg+isoproterenol; IR-C, ischemia reperfusion control; Telmi10 + IR, telmisartan 10 mg/kg + ischemia reperfusion; ABT5 + IR, Abatacept 5 mg/kg + ischemia reperfusion.

To determine the effect of ABT in experimental models of MI, ABT was assessed under two objectives as follows:

1. *Dose optimization using a chemically induced model of myocardial necrosis*: Animals (*n* = 30) were randomly divided into five groups, with six rats in each group. Distilled water was used as a vehicle and administered (2 ml/kg/day) to the rats in normal and ISO-C (Isoproterenol induced control) groups for a period of 21 days, subcutaneously (s.c.). In pretreatment groups, rats were administered with doses of ABT for 21 days, as mentioned in Figure 1. On the 19th and 20th day, animals in group “b–e” were injected with ISO (85 mg/kg; s.c.). On the 21st day, animals were anesthetized with pentobarbitone sodium (60 mg/kg; i.p.). The right carotid artery was cannulated and connected to the transducer to record hemodynamic parameters. Animals were sacrificed using an overdose of anesthesia to obtain heart and blood samples for further evaluation of dose optimization.
2. *Ischemia-reperfusion (IR) injury model of MI:* Animals were divided into five groups (*n* = 6) (Figure 1). An optimized dose of ABT (5 mg/kg) was administered to animals subcutaneously for 21 days in test groups. Telmisartan (a drug used in the treatment of MI) at 10 mg/kg was administered for 21 days in one of the experimental groups, as shown in Figure 1. On the 21st day, animals were subjected to IR injury by surgically blocking the blood flow in the left anterior descending artery for 60 min (ischemia), followed by reperfusion for 45 min. Hemodynamic parameters were recorded during the entire surgical procedure. Postsurgery, animals in all groups were sacrificed using an overdose of pentobarbitone sodium, and the blood, as well as the heart, were collected for further evaluation.

### 2.3. Hemodynamic Parameters

The experimental procedure used for the assessment of hemodynamic and ventricular function was described earlier by Verma et al. [21]. The right carotid artery was cannulated with heparin and saline-filled polypropylene tube (internal diameter 0.39 mm and outer diameter 0.40 mm). The artery was connected with the pressure sensing transducer of the Biopac system (BSL 4.0 MP 36 software) to record hemodynamic variables such as systolic arterial pressure (SAP), mean arterial pressure (MAP), diastolic arterial pressure (DAP), and heart rate (HR). Thereafter, thoracotomy was performed between the fifth and sixth intercostal space using cervical cautery (Delta Medical Appliance, Mumbai, India). A broad perforated sterile metal cannula attached to a pressure sensing transducer was inserted in the left ventricular cavity to record ventricular hemodynamics viz. left ventricular end-diastolic pressure (LVEDP), positive (+LVdP/dt), and negative (−LVdP/dt) left ventricular pressure for the rate of contraction and rate of relaxation, respectively. All hemodynamic parameters were recorded for 10 min in the necrosis model and throughout the surgery in the IR model of MI of rats.

### 2.4. Biochemical Analysis

Snap-frozen part of the heart tissue was thawed at RT (room temperature) and homogenized to make 10% homogenate using ice-chilled phosphate buffer (0.1 M, pH 7.4). The homogenate was divided into three aliquots, of which two aliquots were used for the estimation of malondialdehyde (MDA) and reduced glutathione (GSH) using protocols given by Ohkawa et al. [22] and Moron et al. [23], respectively. The third aliquot was centrifuged at 5,000 rpm for 20 min at 4°C. The supernatant was collected and used for the estimation of superoxide dismutase (SOD) and catalase (CAT) enzyme activities, following the standard protocols Marklund and Marklund [24] and Aebi [25].

### 2.5. Cardiac Injury and Inflammatory Markers

The levels of cardiac injury markers, creatine kinase-myocardial band (CK-MB), Lactate dehydrogenase (LDH), and inflammatory markers, like tumor necrosis factor (TNF) and interleukin-6 (IL-6) were estimated in serum and cardiac tissue (based on the model inflammation) using enzyme-linked immunosorbent assay methods following the manufacturer's protocol.

### 2.6. Histopathological and Immunohistochemical Analysis

The procedure for histopathological studies was used as described previously [26]. For histopathology, the formalin-fixed part of the heart was processed and embedded in paraffin blocks. Then, thin sections of 5–6 *µ*m thickness of cardiac tissues were cut using a microtome (Leica RM 2125, Germany). The sections were stained with hematoxylin and eosin. The sections were mounted and visualized under a light microscope (Dewinter Technologies, Italy) for evaluation of morphological changes. Myocardial damage was scored by a pathologist unaware of the study groups as no change (−), mild (+), moderate (++), and severe (+++).

For the immunohistochemistry, the 5–6 *µ*m thick sections were placed on poly-L-lysin precoated slides. The tissue sections were deparaffinized, and after antigen retrieval, sections were incubated with hydrogen peroxide (30% H_2_O_2_ prepared in methanol) for 30 min to inhibit endo-peroxidase activity. Further, slides were incubated with 10% normal goat serum for 30 min to block the nonspecific binding of collagen and collective tissues. Furthermore, tissue sections were incubated overnight with primary antibodies at 4°C and later with respective HRP-conjugated secondary antibodies. The sections were developed with diaminobenzidine (as the chromogen) counterstained with hematoxylin for 30 s and visualized under a light microscope by a pathologist blinded to the groups of the study.

#### 2.6.1. Terminal Deoxynucleotidyl Transferase dUTP Nick End Labeling (TUNEL) Assay

TUNEL assay was performed as given by Mutneja et al. [27]. Heart sections were incubated with proteinase K for permeabilizing the apoptotic cells. The sections were then incubated with a TUNEL reaction mixture including TdT and Brd-UTP for 60 min following the TUNEL assay kit manufacturer's protocol. Thereafter, the reaction mixture and sections were again incubated with Alexa fluor 488 dye-labeled anti-BrdU antibody for 30 min. After incubation, sections were incubated with propidium iodide solution for 30 min. The TUNEL-positive cells were visualized under a fluorescent microscope (epifluorescence microscope, Nikon, Japan).

### 2.7. Immunoblot Analysis

The procedure followed for immunoblot analysis has been given previously [28], and the proteins such as P38, p-P38, JNK, p-JNK, Erk1/2, p-Erk1/2, Nrf-2, HO-1, Hsp27, Hsp70, NF-*κ*B, p-NF-*κ*B, TGF-*β*, Akt, p-Akt, NOX-4, Cytochrome-C, Bcl_2_, Bax, Caspase 3, GAPDH and *β*-actin were measured using immunoblot. Heart tissue homogenate (10%) in RIPA (radio-immunoprecipitation assay) buffer containing protease inhibitor (Sigma chemicals Co. St. Louis, MO, USA) was prepared and centrifuged at 12,000 rpm for 20 min at 4°C. Using supernatant, total protein concentration was calculated by the Bradford method. An equal amount of reduced protein from different groups was loaded in the SDS–PAGE and resolved. The separated proteins were transferred to the nitrocellulose membrane. The membrane was blocked using 3% bovine serum albumin for 3 hr at RT. Thereafter, the membrane was washed using TBS-T following overnight incubation with primary antibody at 4°C. These primary antibodies were detected by adding HRP-conjugated secondary antibodies for 3 hr at RT. Later, the unbound antibodies were washed thoroughly using TBS-T, and the membrane was visualized with an enhanced chemiluminescence (Thermo Fisher Scientific Inc., USA) kit. However, the housekeeping gene GAPDH was observed using a primary antibody raised in mice and respected anti-mice Alexa Fluro 488 labeled secondary antibody. The specific protein bands were quantified using densitometric analysis through Image J software.

### 2.8. Statistical Analysis

Descriptive data such as mean and standard error of the mean (SEM) was calculated for all variables of each group. One-way analysis of variance, followed by the Tukey comparison post hoc tests, was used to determine the significance using statistical software (GraphPad Prism 10.0.0). The values of *P* ≤ 0.033 ( ^*∗*^), *P* ≤ 0.002 ( ^*∗∗*^), and *P* ≤ 0.001 ( ^*∗∗∗*^), were considered statistically significant following APA (American Physiological Association). While nonsignificant/insignificant is denoted as “ns.”

## 3. Results

### 3.1. ABT Attenuates Inflammation in ISO-Induced Model of Myocardial Necrosis

**Figure 2:**
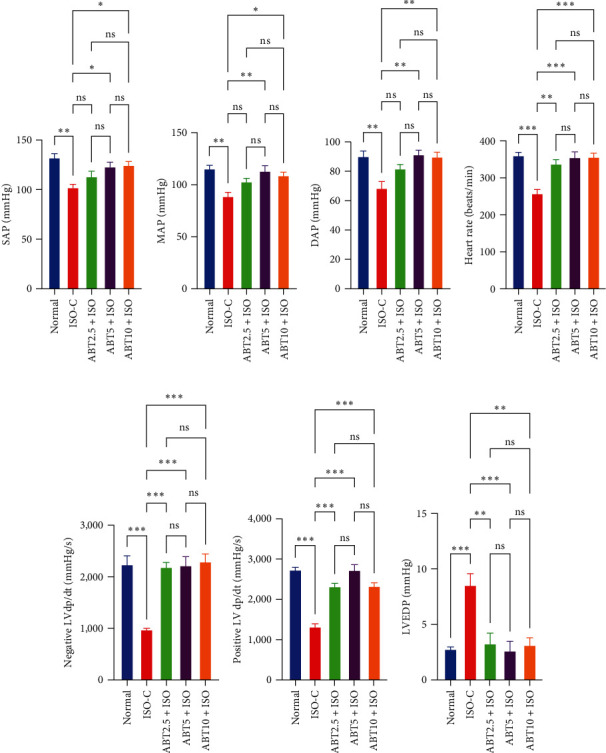
Effect of Abatacept on hemodynamic parameters in ISO-induced model of myocardial necrosis: SAP (a), MAP (b), DAP (c), HR (d), (±) LVdP/dtmax (e and f), and LVEDP (g). Data are expressed as mean ± SEM; *n* = 6 in each group.  ^*∗*^*P* ≤ 0.033,  ^*∗∗*^*P* ≤ 0.002, and  ^*∗∗∗*^*P* ≤ 0.001. SAP, systolic arterial pressure; MAP, mean arterial pressure; DAP, diastolic arterial pressure; HR, heart rate; (+) LVdP/dt, left ventricular (positive) maximal rate of change of pressure; (−) LVdP/dt, left ventricular (negative) maximal rate of change of pressure; LVEDP, left ventricular end-diastolic pressure; ISO-C, isoproterenol control; ABT2.5 + ISO, Abatacept 2.5 mg/kg+isoproterenol; ABT5 + ISO, Abatacept 5 mg/kg + isoproterenol; ABT10 + ISO, Abatacept 10 mg/kg + isoproterenol.

**Figure 3:**
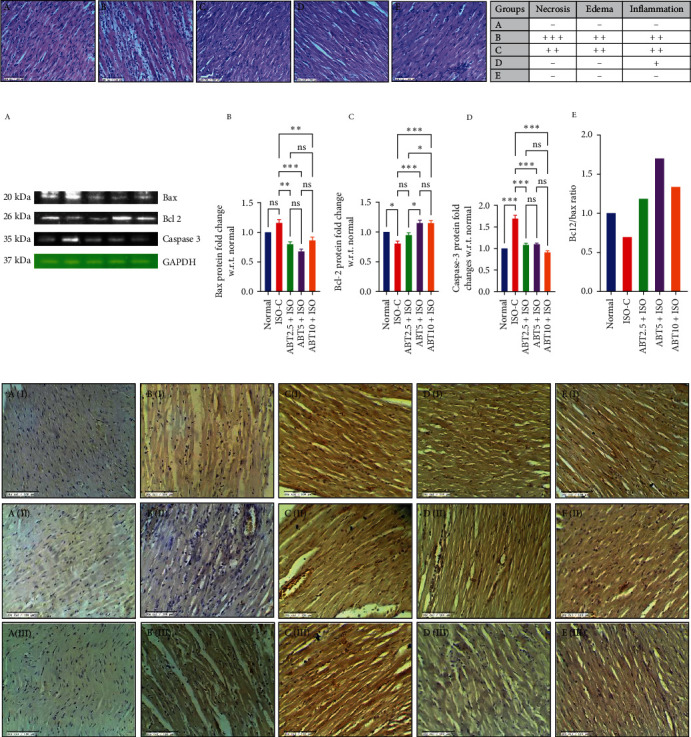
Effect of Abatacept on histopathological changes, apoptosis pathway protein expression and immunohistochemical analysis in ISO-induced rat model of myocardial necrosis. (a) Histopathology (H&E): (*n* = 3, 20x scale bar = 100 *µ*m). Histopathological grading for necrosis, edema, and inflammation: (+++) severe; (++) moderate; (+) mild; (−) no. C (F); (b) apoptosis pathway protein (Bax, Bcl-2, and Caspase-3) expression: data are expressed as mean ± SEM.; *n* = 3 in each group. (c) Effect of Abatacept on immunohistochemical analysis of bax (IA–E), Bcl-2 (IIA–E), and Caspase-3 (IIIA–E); (A) normal; (B) ISO-C; (C) ABT2.5 + ISO; (D) ABT5 + ISO; (E) ABT10 + ISO; (*n* = 3).  ^*∗*^*P* ≤ 0.033,  ^*∗∗*^*P* ≤ 0.002, and  ^*∗∗∗*^*P* ≤ 0.001. ISO-C, isoproterenol control; ABT2.5+ISO, Abatacept 2.5 mg/kg + isoproterenol; ABT5 + ISO, Abatacept 5 mg/kg + isoproterenol; ABT10+ISO, Abatacept 10 mg/kg + isoproterenol.

**Figure 4:**
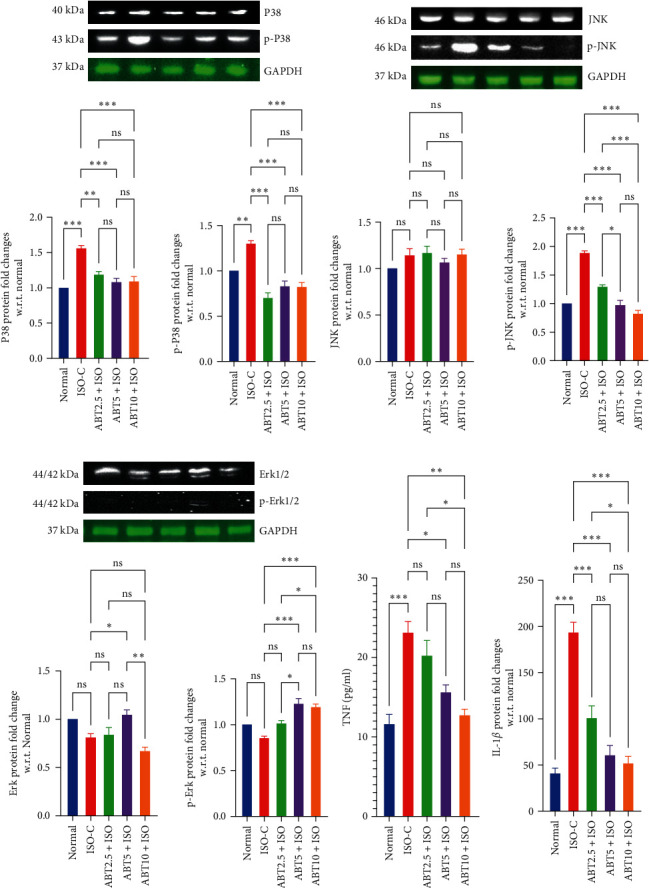
Effect of Abatacept on MAPK signaling protein in ISO-induced rat model of myocardial necrosis. (a) P38/P-P38; (b) JNK/P-JNK; (c) ERK/P-ERK; (d) TNF/IL-6. Data are expressed as mean ± SEM; *n* = 3 in each group.  ^*∗*^*P* ≤ 0.033,  ^*∗∗*^*P* ≤ 0.002, and  ^*∗∗∗*^*P* ≤ 0.001. ISO-C, isoproterenol control; ABT2.5+ISO, Abatacept 2.5 mg/kg+isoproterenol; ABT5+ISO, Abatacept 5 mg/kg+isoproterenol; ABT10+ISO, Abatacept 10 mg/kg + isoproterenol; JNK, c-jun N-terminal Kinase; ERK, extracellular signal-regulated kinase; TNF, tumor necrosis factor; IL-6, interleukin-6.

**Figure 5:**
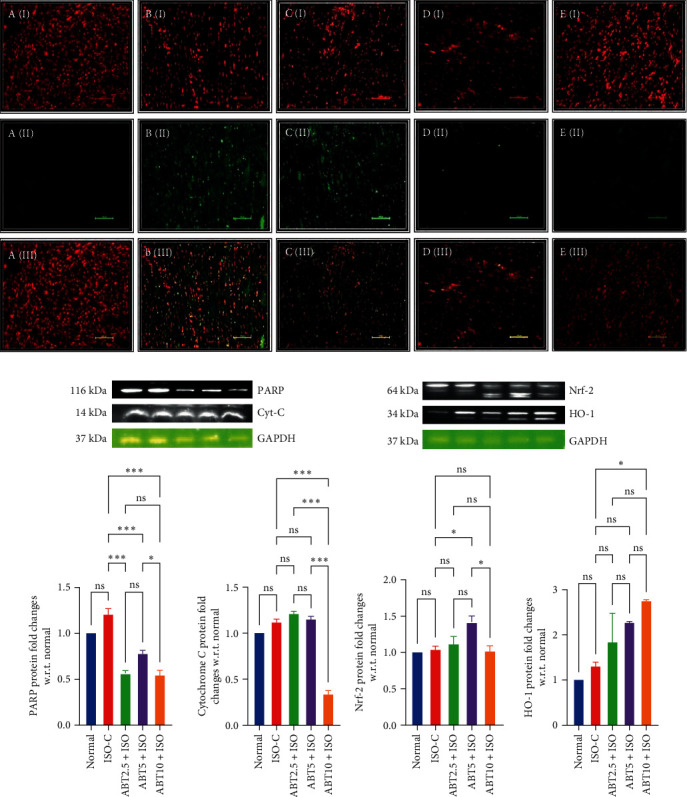
Effect of Abatacept on TUNEL assay in ISO-induced rat model of myocardial necrosis. (a) Protein expression of PARP-cytochrome-C, (b) Nrf−2/HO−1, and (c) in ISO-induced rat model of myocardial necrosis. Data are expressed as mean ± SEM; *n* = 3 in each group.  ^*∗*^*P* ≤ 0.033,  ^*∗∗*^*P* ≤ 0.002, and  ^*∗∗∗*^*P* ≤ 0.001. ISO-C, isoproterenol control; ABT2.5+ISO, Abatacept 2.5 mg/kg+isoproterenol; ABT5+ISO, Abatacept 5 mg/kg+isoproterenol; ABT10+ISO, Abatacept 10 mg/kg+isoproterenol; PARP, poly (ADP-ribose) polymerases; Nrf-2, nuclear factor erythroid 2-related factor 2; HO-1, heme oxygenase-1; TUNEL, terminal deoxynucleotidyl transferase dUTP nick end labeling.

(1) *Hemodynamic parameters in ISO-induced model of myocardial necrosis:* ISO-treated animals (ISO-C group) showed a significant fall in SAP, MAP (*P* ≤ 0.01), DAP, and HR as compared to the normal group (Figure 2(a)–2(d)). ABT pretreatment at all three doses (2.5, 5, 10 mg/kg) showed a significant improvement in blood pressure indices (Figure 2(a)–2(c)). ISO infusion severely affected the ventricular function by the significant reduction in the rate of contraction (+LVdP/dt) and relaxation (−LVdP/dt) phases when compared with the normal group. However, the restoration of ventricular functions was observed with all doses of ABT (Figures 2(e) and 2(f)). Further, a massive rise in LVEDP levels was also observed with ISO administration. Pretreatment with ABT significantly prevented the rise in LVEDP (Figure 2(g)).
(2) *Biochemical parameters in ISO-induced model of myocardial necrosis:* The antioxidant milieu of cardiac tissue was severely disrupted with ISO administration in the ISO-C group. The levels of antioxidants, i.e., GSH, SOD, and CAT, were significantly low, while the levels of MDA were significantly high in the ISO-C group as compared to the normal group (Table 1). ABT pretreatment ameliorated the deficit of endogenous antioxidants in a dose-dependent manner following ISO administration. Through, the improvement in antioxidant levels was observed with ABT but the difference with ABT 2.5 mg/kg was not statistically significant for GSH. Higher doses of ABT, i.e., 5 and 10 mg/kg, not only improved antioxidant levels but also prevented lipid peroxidation (MDA) (Table 1).
(3) *Cardiac injury markers in ISO-induced model of myocardial necrosis:* ISO administration showed tremendous cardiac tissue damage as evidenced by a significant rise in the levels of CK-MB and LDH isoenzyme in serum of the ISO-C group as compared to the normal group. ABT administration at all doses significantly prevented the rise in the levels of CK-MB and LDH. The effect of ABT in alleviating the cardiac injury markers in serum was analogous to the experimental groups (ABT5 + ISO and ABT10 + ISO) (Table 1).
(4) *Pathological changes in ISO-induced model of myocardial necrosis:* The structural architecture of heart tissue was analyzed from hematoxylin-eosin staining as depicted in Figure 3(a). The heart section of rats in the normal group showed no sign of necrosis, edema, and inflammation. ISO-treated myocardium showed marked evidence of necrosis because of which the nucleus from damaged cells was seen accumulating together along with infiltrated blood cells. Also, ISO administration showed marked signs of inflammation and edema. Following ABT pretreatment at 2.5 mg/kg dose, the architecture was improved a bit as the necrosis was less; however, there was evidence of inflammation and edema. At doses of ABT, i.e., 5 and 10 mg/kg, the morphology of the cells appeared similar to the normal group, and the damage was markedly low, based on histopathological scoring (Figure 3(a)).
(5) *Immunohistochemistry in ISO-induced model of myocardial necrosis:* The effect on apoptosis was also assessed in the heart tissues following ABT and ISO administration by apoptotic and antiapoptotic protein expressions, as depicted in Figures 3(b) and 3(c). ISO administration caused higher expressions of Bax and caspase-3 proteins; however, Bcl-2 expression was markedly low when compared to the normal group. With ABT, 2.5 mg dose following ISO administration, the levels of both pro-apoptotic proteins (Bax and caspase-3) and anti-apoptotic protein (Bcl-2) were higher as compared to the normal group. However, with ABT 5 and 10 mg/kg, no change in caspase-3 expression was found between the different dose groups and the normal group when measured through immuno-blot analysis (Figure 3(b)).
(6) *Inflammatory markers in ISO-induced model of myocardial necrosis:* ISO administration caused increased production of pro-inflammatory indicator markers, i.e., TNF and IL−6 in the ISO-C group than that of the normal group. Pretreatment with ABT significantly prevented the expression of these cytokines (TNF and IL-6) at all doses. The cytokines levels in the experimental group (ABT10 + ISO) were found to be statistically similar to the normal group (Figure 4(d)).
(7) *Immunoblot analysis in ISO-induced model of myocardial necrosis*
  - *MAP Kinase Signaling:* ISO administration raised the levels of both p-P38 and p-JNK along with P38; however, no significant changes were observed in Erk1/2 and p-Erk1/2. Treatment with ABT at all doses prevented the rise in the levels of P38 and JNK, with the maximal effect seen at 5 and 10 mg/kg doses. On the other hand, the expression of p-Erk1/2 increased with ABT in a dose-dependent manner, with statistical significance being seen at 5 mg/kg dose. At ABT, 10 mg/kg dosage p-Erk1/2 was reversed significantly in comparison to the ISO-C group (Figure 4(a)–4(c)).
  - *Bax/Bcl-2 Signaling:* Following ISO administration, there was a significant imbalance between Bax and Bcl-2 expressions, thereby affecting downstream signaling to activate caspase-3. ABT 2.5 mg/kg significantly reduced. Bax and caspase-3 levels as compared to the ISO-C group without any effect on Bcl-2 levels. However, ABT 5 mg/kg dose significantly increased the levels of Bcl-2 and decreased the levels of caspase-3. At a 10 mg/kg dose of ABT, there was a significant reversal in the expression of Bax, Bcl-2, and caspase-3 proteins (Figure 3 C2). These changes were confirmed by the Bcl-2/Bax ratio (Figure 3(b): E). Further, on the ISO challenge, the expressions of both Cyt-C and PARP were changed; however, the difference was not statistically significant with Cyt-C (Figure 5(b)). Pretreatment with ABT at all doses significantly reduced the levels of PARP as compared to the ISO-C group.
  - *Nrf-2/HO-1 Signaling:* ISO challenged group did not show any significant changes in Nrf-2 and HO-1 protein expression, whereas treatment with ABT raised the levels of HO-1 significantly in comparison to both normal and ISO-C groups (*P* ≤ 0.001). There was an insignificant difference observed in the Nrf-2 expression in the ABT pretreatment group in comparison to the normal and ISO-C groups (Figure 5(c)).
(8) *TUNEL assay in ISO-induced model of myocardial necrosis:* The antiapoptotic potential of ABT was further confirmed using TUNEL assay, where the number of TUNEL-positive cells was significantly increased in the ISO-C group (Figure 5(a)). These TUNEL-positive cells were shown with green fluorescent signals. ABT pretreatment depicted the maximal improvement at 5 and 10 mg/kg doses, where no green signals were seen. However, no significant effect was witnessed at a 2.5 mg/kg dose of ABT (Figure 5(a)).

### 3.2. ABT Attenuates Inflammation in the IR Injury Model of MI

**Figure 6:**
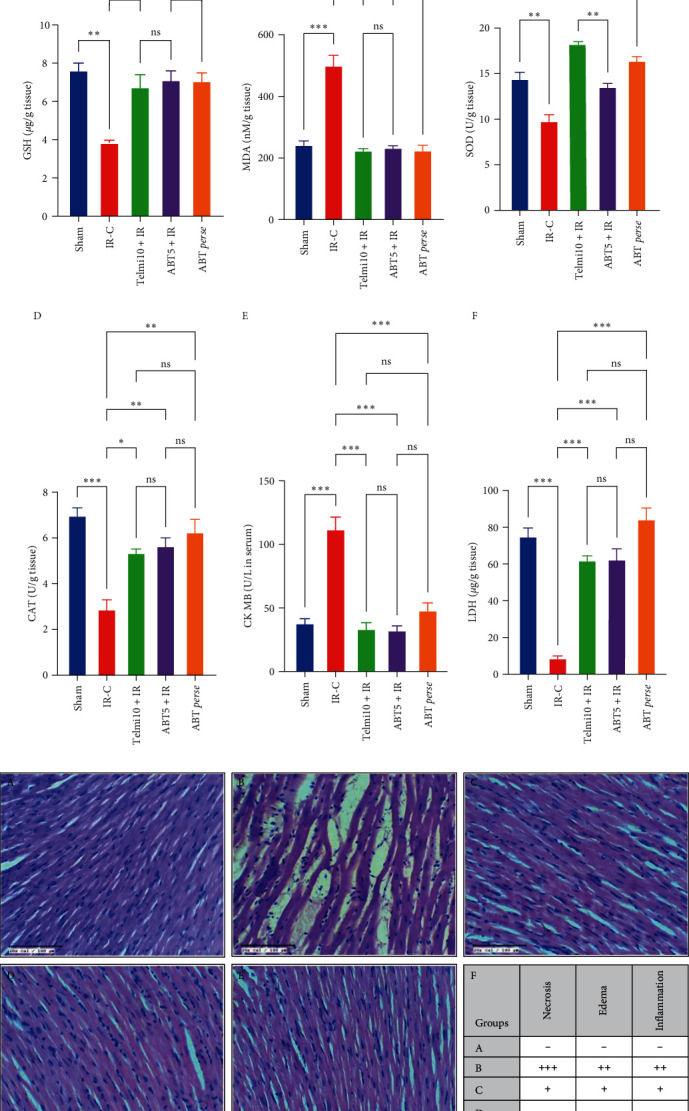
(a) Effect of Abatacept on biochemical parameters in IR injury model; GSH (a-A), MDA (a-B), SOD (a-C), CAT (a-D), and cardiac injury markers CK-MB (a-E), LDH (a-F), data are expressed as mean ± SEM; *n* = 6 in each group. (b) Tissue histopathology with scoring (A–F) in ischemia-reperfusion (IR) injury model of myocardial infarction. (A) Normal; (B) IR-C, (C) Telmi10+IR, (D) ABT5 + IR, (E) ABT *perse*. (F) Grading for necrosis, edema, and inflammation: (+++) severe; (++) moderate; (+) mild; (−) no.  ^*∗*^*P* ≤ 0.033,  ^*∗∗*^*P* ≤ 0.002, and  ^*∗∗∗*^*P* ≤ 0.001. GSH, glutathione; MDA, malondialdehyde; SOD, superoxide dismutase; CAT, chloramphenicol acetyltransferase; CK-MB, creatine kinase-myocardial band; LDH, lactate dehydrogenase; IR-C, ischemia-reperfusion control; Telmi10 + IR, telmisartan 10 mg/kg + ischemia-reperfusion; ABT5 + IR, Abatacept 5 mg/kg + ischemia-reperfusion.

**Figure 7:**
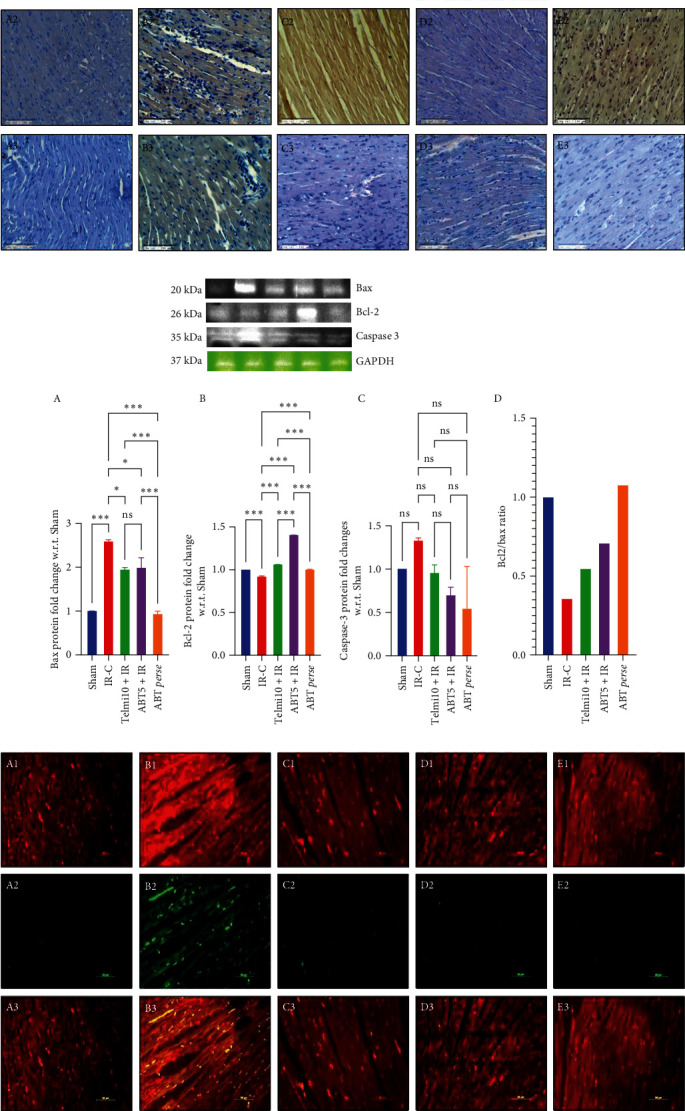
Effect of Abatacept on Apoptosis in IR injury model of myocardial infarction. (a) Immunohistochemical analysis of bax, Bcl−2 and Caspase−3; (b) immuno-blot analysis of bax, Bcl-2, Caspase-3, and Bcl-2/Bax ratio. (c) TUNEL assay. Data are expressed as mean ± SEM; *n* = 3 in each group.  ^*∗*^*P* ≤ 0.033,  ^*∗∗*^*P* ≤ 0.002, and  ^*∗∗∗*^*P* ≤ 0.001. IR-C, ischemia-reperfusion control; Telmi10+IR, telmisartan 10 mg/kg+ischemia reperfusion; ABT5+IR, Abatacept 5 mg/kg+ischemia reperfusion; TUNEL, terminal deoxynucleotidyl transferase dUTP nick end labeling.

**Figure 8:**
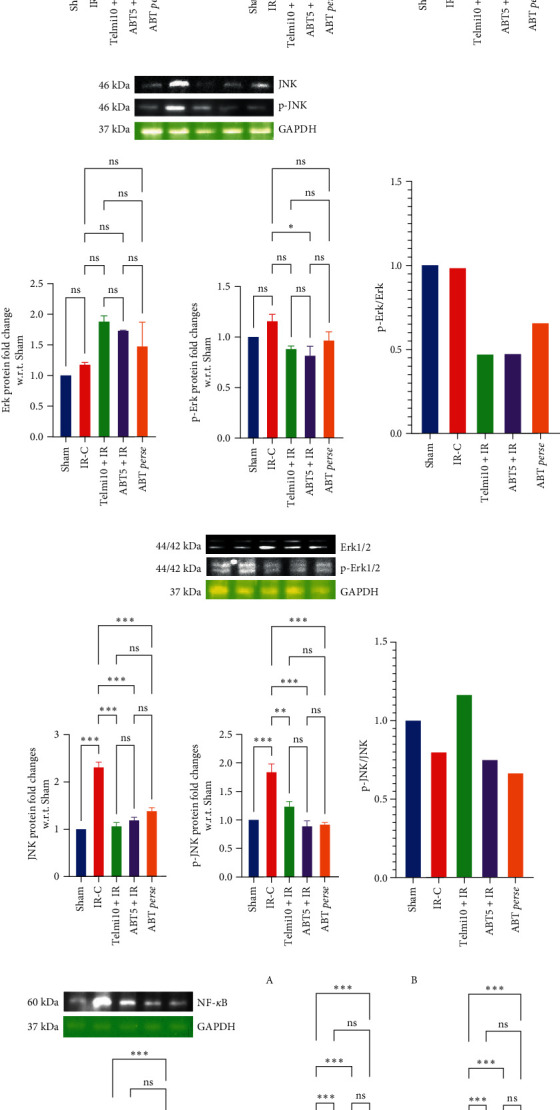
Effect of Abatacept on MAPK signaling pathway proteins in IR injury model of myocardial infarction. (a) P38, p-P38, and their ratio (p-P38/P38); (b) JNK, p-JNK, and their ratio (p-JNK/JNK); (c) ERK 1/2/P-ERK 1/2; (d) downstream pathway NF-*κ*B; (e) cytokine levels [TNF/IL-6 (e: A–B)]. Data are expressed as mean ± SEM; *n* = 3 in each group. IR-C, ischemia-reperfusion control; Telmi10+IR, telmisartan 10 mg/kg+ischemia reperfusion; ABT5+IR, Abatacept 5 mg/kg+ischemia reperfusion; MAPK, mitogen-activated protein kinase; JNK, c-jun N-terminal Kinase; ERK, extracellular signal-regulated kinase; NF-*κ*B, nuclear factor kappa B; TNF, tumor necrosis factor, IL-6: interleukin-6.

**Figure 9:**
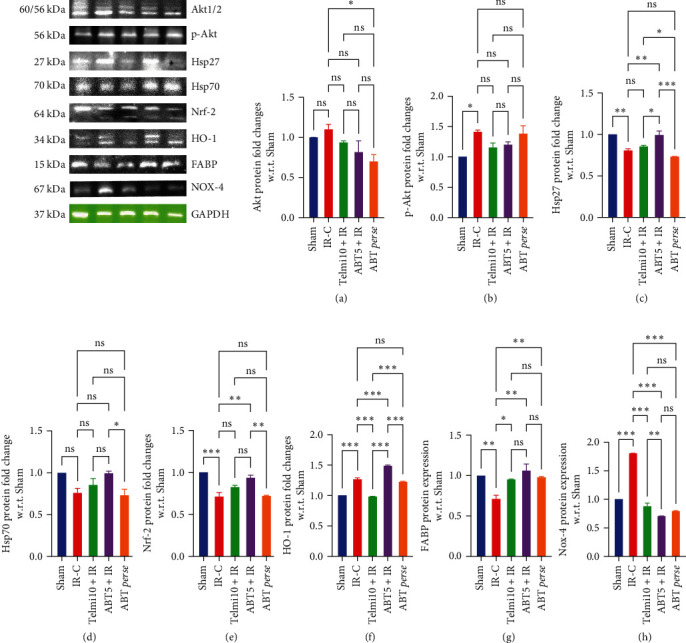
Effect of Abatacept on Akt1/2/p-Akt (a) and (b); Hsp (27, 70) (c) and (d); Nrf-2/HO-1 (e) and (f); FABP (g); and NOX-4 (h) in ischemia-reperfusion (IR) injury model of myocardial infarction. Data are expressed as mean ± SEM; *n* = 3 in each group.  ^*∗*^*P* ≤ 0.033,  ^*∗∗*^*P* ≤ 0.002, and  ^*∗∗∗*^*P* ≤ 0.001. IR-C, ischemia reperfusion control; Telmi10+IR, telmisartan 10 mg/kg+ischemia reperfusion; ABT5+IR, Abatacept 5 mg/kg+ischemia reperfusion; Hsp, heat shock protein; Nrf-2, nuclear factor erythroid 2-related factor 2; HO-1, heme oxygenase-1; FABP, fatty acid binding proteins; NOX-4, NADPH oxidase 4.

(1) *Hemodynamic parameters in IR injury model:* The hemodynamic parameters were observed continuously during the ischemia (60 min) and reperfusion (45 min) phases. At baseline, MAP in all groups was similar in the range of 92.5 ± 1.8 to 95.9 ± 0.7 mmHg. Immediately after the onset of ischemia, the MAP was significantly reduced in all groups as compared to the Sham group except ABT *perse*. After 30 min of the ischemic phase, MAP was significantly improved in both ABT5 + IR and Telmi10 + IR pretreated groups compared to the IR-C group. Though the blood pressure was improved at reperfusion zero minute in ABT and telmisartan pretreated groups, the difference was insignificant in the IR-C group and ABT5 + IR group. At the end of the experiment, i.e., reperfusion 45 min, significant improvement was observed with both ABT and telmisartan pretreated groups experiencing IR, and their MAP values were close to the MAP values of the Sham group. Furthermore, the HR was significantly reduced in the IR-C group; however, ABT and telmisartan pretreatment significantly preserved heart function even after ischemia induction. The improvement in HR was continued till the end of the experimental hemodynamic monitoring in the ABT5 + IR and Telmi10 + IR groups (Table 2). In addition to this, left ventricular functions were recorded and showed a significant fall in +LVdP/dt and −LVdP/dt and an increase in LVEDP during IR. At all-time points, from the induction of ischemia till the end of reperfusion, the ventricular functions were effectively restored with the pretreatment of ABT and telmisartan. During IR injury in the ABT5+IR and Telmi10+IR groups, the levels of ±LVdP/dt and LVEDP were significantly increased and decreased, respectively, as compared to the IR-C. In the ABT *perse* group, no such ventricular function changes were observed at any time point during the procedure (Table 2).
(2) *Biochemical Parameters in IR injury model:* IR-challenged myocardium resulted in marked elevation of tissue MDA, along with a fall in the levels of GSH, SOD, and CAT (Figure 6(a)). Intriguingly, Telmi10 + IR, ABT5 + IR, and ABT *perse* showed impressive and statistically similar MDA levels as compared to the Sham group. Pretreatment with ABT significantly enhanced the levels of antioxidants, i.e., GSH, SOD, and CAT, even after IR injury in ABT5 + IR groups which were higher than that of the Sham group (Figure 6(a): A–D).
(3) *Cardiac Injury Markers in IR injury model:* The IR injury-induced cardiac damage was shown with a significant rise in the levels of CK-MB in serum and a drop in LDH levels in cardiac tissue in the IR-C group as compared to the Sham group. However, ABT pretreatment at 5 mg/kg dose significantly prevented the cardiomyocyte damage and release of CK-MB and LDH into circulation when compared to the IR-C group. Similarly, in Telmi10 + IR and ABT *perse* groups, the levels were similar to the Sham group (Figure 6(a): E and F).
(4) *Pathological Changes in IR injury model:* The leakage of cardiac injury markers in the bloodstream was further noticed with the assessment of the histoarchitecture of myocardial cells as depicted in Figure 6(b). Following IR injury in the IR-C group, marked edema, membrane damage, and infiltration of inflammatory cells, along with necrosis and inflammation, were observed. In contrast, ABT pretreatment restricted myonecrosis, preserved the cardiomyocyte membrane, minimized the infiltration, and exhibited a low pathological score (Figures 6(b) and 6(b: F)).
(5) *Immunohistochemistry in IR injury model:* To study the role of ABT on apoptosis following IR injury, immunohistochemistry was performed, and expression of pro- and anti-apoptotic proteins was compared in all groups. In the IR-C group, a significant increase in Bax expression was noticed, while no sign of Bcl-2 and caspase-3 was seen. Intriguingly, ABT pretreatment at 5 mg/kg increased the Bcl-2 expression. The balance between Bax and Bcl-2 expression prevented the activation of caspase-3, and hence, no expression of caspase-3 was observed in the ABT5 + IR group. Similarly, in the telmisartan group, no increased expression of Bax and caspase-3 was observed rather than increased levels of Bcl-2. In the ABT *perse* group, no significant change was observed in either protein expression (Figure 7(a)).
(6) *Inflammatory Markers in IR injury model:* IR injury-induced inflammatory response was estimated by observing the levels of TNF and IL-6 in blood serum. In IR-C rats, marked increases in the levels of pro-inflammatory markers were observed w.r.t. Sham group. This inflammatory response following IR injury was completely inhibited in the ABT5 + IR and Telmi10 + IR groups. The levels of TNF and IL-6 in the IR-C group were significantly restored following ABT pretreatment, which was comparable to the Sham group (Figure 8(e): A and B).
(7) *Immunoblot Analysis in IR injury model:*
  - *MAP kinase Signaling*: Following IR injury, MAPK proteins, including p-P38, JNK, and p-JNK, were significantly raised in the IR-C group as compared to the sham group. Pretreatment with ABT 5 mg/kg markedly reduced the levels of p-P38 even less than that of the Sham group without any significant changes in P38 protein expression. Moreover, ABT administration also restores the JNK and p-JNK levels even after IR injury, and the levels were also parallel to the Telmi10 + IR group. Furthermore, levels of Erk1/2 were raised in both telmisartan and ABT pretreatment without any marked changes in p-Erk1/2 expression (Figure 8(a)–8(c)). IR injury caused a significant rise in the levels of NF-*κ*B as compared to the Sham group. ABT pretreatment at 5 mg/kg significantly reversed the levels of NF-*κ*B (Figure 8(d)).
  - *Bax/Bcl-2 Signaling*: The IR-C group exhibited a significant rise in the levels of Bax and caspase-3 without any significant changes in Bcl-2 levels in comparison to the sham group. Pretreatment with ABT at 5 mg/kg not only reduced the levels of Bax and caspase-3 but also enhanced the expression of Bcl-2 as compared to the IR-C group. Whereas treatment with telmisartan at 10 mg/kg did not show such improvement, as the expressions of Bax were high as compared to the Sham group; however, the levels of caspase-3 were similar to Sham (Figure 7(b)). To see the trend, the Bcl−2/Bax ratio was assessed, and maximal protection with ABT 5 mg/kg group following IR injury was observed (Figure 7(b)).
  - *Nrf-2/HO-1 Signaling:* IR injury caused a significant fall in the levels of Nrf-2, Hsp27, and Hsp70 as compared to the Sham group. However, pretreatment with ABT showed significantly high levels of Nrf-2 and HO-1 (Figures 9(e) and 9(f)) along with co-antioxidant molecules such as Hsp27 and Hsp70 (Figures 9(c) and 9(d)) as compared to IR-C group, which were also statistically significant with the Sham group. On the other hand, no such changes were observed with the Telmi10 + IR group.
  - *Akt/p-Akt Signaling:* Following IR injury, an immediate rush in the levels of both Akt and p-Akt was observed; hence, the difference was not statistically significant, whereas ABT pretreatment reduced the levels of Akt in ABT5 + IR and ABT *perse* groups as compared to the IR-C group (Figures 9(a) and 9(b)).
  - *FABP and NOX-4 Expressions:* The decreased expression of FABP in the IR-C group was significantly improved with ABT and telmisartan pretreated animals (Figure 9(g)). IR injury caused a significant rise in the levels of NOX-4 as compared to the Sham group. Pretreatment with ABT at 5 mg/kg and telmisartan at 10 mg/kg significantly reversed the levels of NOX-4 (Figure 9(h)).
(8) *TUNEL Assay in IR injury model:* The role of ABT in apoptosis was further confirmed by the TUNEL assay, where a significantly increased number of TUNEL-positive cells was found in the IR-C group as compared to the Sham group. Following ABT treatment, the number of TUNEL-positive cells was markedly decreased as compared to the IR-C group, and the expression was found to be similar to that of Sham. No sign of apoptosis was observed in the ABT *Perse* group (Figure 7(c)).

## 4. Discussion

In the present study, we demonstrated the novel role of ABT in combating ISO-induced cardiac injury by improving hemodynamic parameters, cardiac tissue injury, and inflammation in rats. The MI triggers the altered shape and size of the ventricle, known as “ventricular remodeling” which remains a major pathological mechanism for chronic heart failure following MI [29]. The ventricular remodeling also involves the alteration in the myocardial cells along with the ECM. The dysregulated balance between the production and lysis of collagen in the ECM following MI leads to fibrosis, resulting in fewer myocardial activities and a decrease in myocardial contractility and ventricular compliance, ultimately leading to heart failure [30]. The foremost observation in the present study is that pretreatment with ABT at all doses significantly restored blood pressure indices, viz. systolic, diastolic, and MAP, along with HR, as compared to the ISO-C group. It offered maximum restoration of LVEDP and ±LVdP/dt parameters, thus showing improved ventricular function. Our results are in resonance with the previously published study by Kallikourdis et al. [31], demonstrating a significant reduction in ventricular preload with ABT treatment and thus preventing the severity and progression of cardiac dysfunction.

Further, administration of ISO is well known to exaggerate the demand for myocardial oxygen by increasing both HR and contractility via stimulation of *β*-adrenoceptors resulting in dysregulation of the physiological oxidant/antioxidant balance, leading to enhanced lipid peroxidation and diminished antioxidant enzymes [32, 33]. ISO administration also increases ROS production, leading to myocardial damage during MI [34]. The damaged myocardium releases CK-MB, CK, aspartate aminotransferase, LDH, and cardiac troponin I and C into the blood, and the evaluation of these biochemical parameters represents the myocardial injury [33]. Our study showed that the pretreatment with 5 and 10 mg/kg doses of ABT markedly improved the levels of antioxidants, including GSH, SOD, and CAT, following the ISO challenge. The previously published study comments on the antioxidant potential of ABT and also explains that ROS production is attributed to the infiltration of T cells at the site of injury, even in the heart [34]. Hence, inhibition of T-cell activation by ABT might be hypothesized to decrease oxidative stress indirectly. This indirect correlation between T-cell inhibition by ABT and reduced oxidative stress might be hypothesizing that ABT has antioxidant potential. Further, our study also showed that pretreatment with ABT significantly decreased lipid peroxidation (MDA) and leakage of cardiac enzymes, i.e., CK-MB and LDH, after ISO administration and in IR injury.

In addition, ABT improved the histoarchitecture of cardiac tissues when subjected to ISO-induced and IR injury. ABT not only prevented the alterations in the morphology of myocardial cells but also contributed to the inhibition of cell death response. This was observed with the TUNEL assay, where an ABT 5 mg/kg dose markedly reduced the number of TUNEL-positive cells. ABT maintains the levels of Bax and Bcl-2 due to the maintained equilibrium between these two proteins that modulate the downstream pathway for activation of caspase-3 [35, 36].

Furthermore, MI-mediated myocardial damage comprises several signaling molecules. In the ISO-administered MI model, the activation of NF-*κ*B has been reported, which is known as a key regulator of inflammation, necrosis, and fibrosis [37]. Also, administration of ISO causes the phosphorylation of NF-*κ*B, which in turn initiates the intracellular signaling pathway, leading to the upregulation of pro-inflammatory cytokines such as TNF, IL-6, IL-1*β*, as well as cardiac-specific markers [38]. It has been reported that following MI, cytokines such as TNF and IL-6 contribute to the formation of collagen and thereby induce the formation of scars [39]. Elevated levels of IL-6 following MI are reported to be interlinked with the size and severity of the infarct area [40]. Therefore, we have investigated the levels of cytokines in our study. We found that pretreatment with ABT significantly prevented the ISO-induced and IR-injury-related increase in the levels of TNF and IL-6 in serum. Our results corroborate a previous study that reported the anti-inflammatory effect of ABT in RA [41]. Hence, ABT-mediated inhibition of T-cell infiltration into the myocardium could lead to decreased ECM modulation, cardiac remodeling, and inflammatory response [31, 42].

To decipher the molecular signaling pathway, eliciting the probable effect of ABT in MI models, we found that the anti-inflammatory effect of ABT occurs through the modulation of MAP kinase signaling. Its antioxidant potential was elucidated through the activation of the Nrf-2/HO-1 pathway, while the antiapoptotic effect was mediated through the Bax/Bcl-2/caspase-3 pathway. In view of the above-mentioned results, we concluded that ABT 5 mg/kg dose is optimal with maximum efficacy and potency in a murine model of ISO-induced myocardial necrosis.

Further, the optimal dose of ABT 5 mg/kg in the IR model, not only normalized the arterial pressure and HR but also ventricular functions in rats. Marked protection of all hemodynamic parameters was seen instantly after the onset of ischemia in the ABT-administered group. Also, the effect of ABT on cardiac functions and hemodynamics was comparable to the telmisartan pretreated group. Telmisartan administration also showed a significant reversal of both hemodynamic and ventricular functions, along with the prevention of cardiac enzyme leakage. Though the effect of ABT has not been reported before, observations with telmisartan pretreatment were in line with the previous studies [43].

Since ROS generation during reperfusion leads to cellular damage in the myocardium, DNA fragmentation, and ultrastructural changes, we further evaluated the antioxidant role of ABT in IR [44]. In the present study, a drastic imbalance between oxidant and antioxidant levels was observed, thereby causing the activation of cell death pathways. The levels of antioxidants in ABT pretreated groups resemble to standard drug telmisartan, emphasizing the potential antioxidant property of ABT. Similar to our finding, a study done by Huang and Song [45] showed a protective effect of nano-formulation of ABT against lupus nephritis through its antioxidative and anti-inflammatory properties. In addition, pretreatment with both ABT and telmisartan significantly inhibited the initiation of the inflammatory cascade. Previous studies have delineated possible mechanisms behind the anti-inflammatory effect of telmisartan through the activation of PPAR-*γ* [46, 47].

Oxidative stress also led to the induction of apoptosis in IR injury [48]. In this study, IR-C rats showed marked signs of DNA fragmentation in TUNEL assay along with increased levels of pro-apoptotic proteins, i.e., Bax and caspase-3, in contrast to ABT pretreated groups where increased levels of anti-apoptotic protein, i.e., Bcl-2 was observed. There is enough evidence to prove the anti-apoptotic potential of ABT [49, 50].

Moreover, the in-depth analysis showed the anti-inflammatory effect of ABT via inhibition of MAP kinase signaling where ABT significantly decreased the phosphorylation of JNK and P38 along with p-Erk1/2, ultimately leading to downregulation of NF-*κ*B, which is the downstream molecule of MAPK. Previous studies have shown that the MAPK pathway is imperative for cell survival and apoptosis and the involvement of MAPK/NF-*κ*B pathway in myocardial IR injury in vivo and in vitro [51, 52]. MI/R causes an increase in cytokines such as TNF, IL-1*β*, and IL-6. Also, myocardial infarct increases cell permeability, leads to a caspase cascade reaction; subsequently, apoptosis of myocardial cells occurs. Our results showed that ABT reduces the levels of TNF and IL-6. Plethora of studies reported that the inhibition of MAPK activation can activate Nrf-2 and then the regulation of HO-1 [53, 54]. HO-1 plays an essential role in the prevention of injury caused by MIRI. Our experimental results confirmed that the levels of Nrf-2, HO-1, and SOD were decreased, and MDA was increased in the MI/R model as compared to the sham group. ABT treatment increased the levels of Nrf-2, HO-1, and SOD and decreased the level of MDA. In addition, the antioxidant potential of ABT was observed through the modulation of Nrf-2/HO-1 proteins along with their downstream signaling molecules, including Hsp27 and Hsp70. Though the effect of ABT on Akt/p-Akt signaling is controversial, its cardioprotective effect was evidenced with FABP, NOX-4, and NF-*κ*B levels similar to the previous studies [55, 56]. Thus, our findings suggest that ABT could be used as a cardioprotective agent by ameliorating oxidative stress, apoptosis, and inflammation.

## 5. Conclusion

The findings of our study highlight that ABT ameliorates MI by anti-inflammatory and antioxidative effects via inhibiting MAP kinase signaling and modulating Nrf-2/HO-1 proteins downstream signaling cascade, respectively. Hence, ABT could be a valuable drug for alleviating myocardial damage in MI patients. ABT's pharmacokinetic, pharmacodynamic, and safety profiles make it an ideal candidate for the treatment of CVDs. The study also suggests that ABT could be used as a preventive measure or as part of post-secondary prevention in patients at high-risk substantial risk for developing CVD such as MI, etc., or to reduce the severity of preexisting CVD. However further research needs to be elucidated to validate the long-term safety and efficacy of ABT.

## Figures and Tables

**Table 1:** Biochemical evaluation of oxidant-antioxidant and cardiac injury markers in ISO induced model of myocardial necrosis.

Groups	GSH (mg/g of tissue)	MDA (*µ*M/g of tissue)	SOD (U/mg of protein)	CAT (U/ml)	CK-MB (U/l)	LDH (U/l)
Normal	6.03 ± 0.62	0.34 ± 0.03	12.48 ± 0.63	6.28 ± 0.45	350.3 ± 17.15	323.8 ± 15.20
ISO-C	3.19 ± 0.38 ^*∗*^	0.62 ± 0.02 ^*∗∗∗*^	6.38 ± 0.52 ^*∗∗∗*^	2.15 ± 0.61 ^*∗∗∗*^	698.2 ± 47.63 ^*∗∗∗*^	701.9 ± 53.17 ^*∗∗∗*^
Abata2.5 + ISO	4.90 ± 0.73	0.48 ± 0.04^#^	7.53 ± 0.38	4.61 ± 0.38^#^	491.9 ± 35.73^###^	605.2 ± 52.38^###^
Abata5 + ISO	5.82 ± 0.36^#^	0.41 ± 0.02^###^	10.10 ± 0.46^###^	5.2 ± 0.51^##^	372.6 ± 20.62^###^	498.3 ± 19.52^###^
Abata10 + ISO	6.1 ± 0.82^#^	0.39 ± 0.03^###^	11.48 ± 0.62^###^	5.8 ± 0.31^###^	341.3 ± 23.46^###^	443.4 ± 21.30^###^

^*∗*/#^*P* < 0.05,  ^*∗∗*/##^*P* < 0.01,  ^*∗∗∗*/###^*P* < 0.001. ISO-C, isoproterenol control; ABT2.5+ISO, Abatacept 2.5 mg/kg+isoproterenol; ABT5+ISO, Abatacept 5 mg/kg+isoproterenol; ABT10+ISO, Abatacept 10 mg/kg + isoproterenol; GSH, glutathione; MDA, malondialdehyde; SOD, superoxide dismutase; CAT, catalase; CK-MB, creatine kinase−myocardial band; LDH, lactate dehydrogenase.

**Table 2:** Effect of Abatacept on hemodynamic parameters (MAP, HR, LVEDP, (±) LVdP/dt) at various time intervals in ischemia-reperfusion (IR) injury model of myocardial infarction.

Groups	MAP (mmHg)	Heart rate (bpm)	LVEDP	Positive LVdP/dt (mmHg/s)	Negative LVdP/dt (mmHg/s)
Baseline (0 min)					
Sham	92.6 ± 2.0	375.3 ± 3.4	3.2 ± 0.1	2,100.9 + 18.9	1,787.5 ± 12.7
IR-C	92.5 ± 1.8	374.7 ± 1.4	3 ± 0.1	2,110.5 ± 24.6	1,795.2 ± 7.8
Telmi10 + IR	95.1 ± 1.3	375.5 ± 3.1	3.4 ± 0.1^#^	2,081.2 ± 16.2	1,782.9 ± 12.4
ABT5 + IR	95.9 ± 0.7	377.2 ± 2.1	3.1 ± 0.1	2,104.3 ± 15.1	1,796.8 ± 10.4
ABT *perse*	95.6 ± 0.6	374.5 ± 2.9	2.9 ± 0.1	2,061.5 ± 26.5	1,784.8 ± 8.6
Ischemia (0 min)					
Sham	89.7 ± 1.7	378 ± 2.1	3.2 ± 0.1	2,101.7 ± 21.3	1,780 ± 12.8
IR-C	68.4 ± 1.6 ^*∗∗∗*^	292.7 ± 2.1 ^*∗∗∗*^	6.3 ± 0.1 ^*∗∗∗*^	1,864.6 ± 17.8 ^*∗∗∗*^	1,578 ± 31.1 ^*∗∗∗*^
Telmi10 + IR	74.8 ± 1.4 ^*∗∗∗*^^/#^	334.6 ± 4.3 ^*∗∗∗*^^/###^	4 ± 0.2 ^*∗∗*^^/###^	1,978.6 ± 15.2 ^*∗∗*^^/##^	1,686 ± 18.7 ^*∗*^^/#^
ABT5 + IR	75.7 ± 0.9 ^*∗∗∗*^^s/##^	327.9 ± 3.3 ^*∗∗∗*^^/###^	3.7 ± 0.1^###^	1,960.4 ± 11.9 ^*∗∗∗*^^/#^	1,714.6 ± 28^##^
ABT *perse*	87.4 ± 0.5^###/$$$^	369.4 ± 4^###/$$$^	3 ± 0.1^###/$$$^	2,095 ± 27.2^###/$$^	1,766.2 ± 10.6^###^
Ischemia (30 min)					
Sham	89.2 ± 2.1	370 ± 2.4	3.2 ± 0.1	2,076.5 ± 23.7	1,765.8 ± 13
IR-C	70.7 ± 2.0 ^*∗∗∗*^	302.2 ± 2.9 ^*∗∗∗*^	6.6 ± 0.1 ^*∗∗∗*^	1,775 ± 16.2 ^*∗∗∗*^	1,581.2 ± 14.7 ^*∗∗∗*^
Telmi10 + IR	77.7 ± 1.2 ^*∗∗∗*^^/#^	349.1 ± 3.2 ^*∗∗*^^/###^	4.5 ± 0.2 ^*∗∗∗*^^/###^	1,962 ± 13.6 ^*∗∗*^^/###^	1,642.8 ± 22.3 ^*∗∗∗*^
ABT5 + IR	78.4 ± 0.7 ^*∗∗∗*^^/##^	350.8 ± 4.4^###^	4.1 ± 0.1 ^*∗∗∗*^^/###^	1,949.4 ± 13.9 ^*∗∗*^^/###^	1,691.4 ± 21.3 ^*∗*^^/###^
ABT *perse*	88.6 ± 0.7^###/$$$^	363.3 ± 4.5^###^	3 ± 0.1^###/$$$^	2,039.3 ± 31.5 ^###^	1,769.9 ± 9.2^###/$$$^
Ischemia (60 min)/reperfusion (0 min)					
Sham	90.2 ± 2.2	370.5 ± 1.8	3.3 ± 0.1	2,068.4 ± 22.5	1,739.1 ± 13.3
IR-C	74.4 ± 2.3 ^*∗∗∗*^	320.4 ± 2.4 ^*∗∗∗*^	6.9 ± 0.1 ^*∗∗∗*^	1,817.6 ± 20.8 ^*∗∗∗*^	1,531.1 ± 28.3 ^*∗∗∗*^
Telmi10 + IR	82.7 ± 0.9 ^*∗*^^/##^	318.7 ± 4.3 ^*∗∗∗*^	5 ± 0.1 ^*∗∗∗*^^/###^	1,961.3 ± 14.6 ^*∗∗*^^/###^	1,590.3 ± 17.5 ^*∗∗∗*^
ABT5 + IR	79.7 ± 0.9 ^*∗∗∗*^	326.4 ± 2.8 ^*∗∗∗*^	4.6 ± 0.1 ^*∗∗∗*^^/###^	1,933.3 ± 11.1 ^*∗∗∗*^^/##^	1,621.6 ± 21.1 ^*∗∗*^^/#^
ABT *perse*	89.7 ± 0.6^###/$^	361 ± 4.2^###/$$$^	3 ± 0.1^###/$$$^	2,030.5 ± 25.5^###^	1,774.7 ± 8.8^###/$$$^
Reperfusion (45 min)					
Sham	86.4 ± 2.2	365.4 ± 1.7	3.3 ± 0.1	2,064.8 ± 21.6	1,732.6 ± 13
IR-C	71.2 ± 2.0 ^*∗∗∗*^	268.4 ± 0.9 ^*∗∗∗*^	7.2 ± 0.1 ^*∗∗∗*^	1,757.8 ± 20.2 ^*∗∗∗*^	1,451.8 ± 16.7 ^*∗∗∗*^
Telmi 10 + IR	78.3 ± 0.8 ^*∗∗*^^/#^	321.6 ± 3 ^*∗∗∗*^^/###^	5.4 ± 0.1 ^*∗∗∗*^^/###^	1,970.8 ± 22 ^*∗*^^/###^	1,576.8 ± 18.2 ^*∗∗∗*^^/###^
ABT5 + IR	77.4 ± 1.2 ^*∗∗*^^/#^	317.9 ± 1.8 ^*∗∗∗*^^/###^	5.1 ± 0.1 ^*∗∗∗*^^/###^	1,911.5 ± 13.9 ^*∗∗∗*^^/###^	1,589.5 ± 17.4 ^*∗∗∗*^^/###^
ABT *perse*	80.6 ± 0.6^##^	360.8 ± 3.1^###/$$$^	3 ± 0.1^###/$$$^	2,009.8 ± 28.8^###^	1,776.5 ± 9^###/$$$^

^*∗*/#/$^*P* < 0.05,  ^*∗∗*/##/$$^*P* < 0.01,  ^*∗∗∗*/###/$$$^*P* < 0.001. IR-C, ischemia reperfusion control, Telmi10+IR, telmisartan 10 mg/kg+ischemia reperfusion; ABT5+IR, Abatacept 5 mg/kg+ischemia reperfusion.

## Data Availability

The raw data generated during the study will be available from the corresponding author upon reasonable request.

## Additional Points

*Highlights*. This is the first report of Abatacept showing its cardio-protective effect. Abatacept is primarily used in the treatment of rheumatoid arthritis (RA). However, RA patients have a twofold increased risk of myocardial infarction. This study explains the mechanistic role of Abatacept in murine models of myocardial infarction, along with other supporting parameters. This study outcome gives evidence that the use of Abatacept in RA patients may even decrease the risk of myocardial infarction. Findings from this study also give ideas to other researchers to explore the role of Abatacept in different diseases and further clinical trials.

## Supplementary Materials

Supplementary 1 Supplementary 1 shows the detailed list of primary antibodies.

Supplementary 2 Supplementary 2 shows the details of ELISA kits used in the experiments.

## References

[1] WHO (2023). *Cardiovascular Diseases (CVDs)*.

[2] Hu Y., Lu H., Li H., Ge J. (2022). Molecular basis and clinical implications of HIFs in cardiovascular diseases. *Trends in Molecular Medicine*.

[3] Saleh M., Ambrose J. A. (2018). Understanding myocardial infarction. *F1000Research*.

[4] Libby P., Buring J. E., Badimon L. (2019). Atherosclerosis. *Nature Reviews Disease Primers*.

[5] Beijk M. A., Vlastra W. V., Delewi R. (2019). Myocardial infarction with non-obstructive coronary arteries: a focus on vasospastic angina. *Netherlands Heart Journal*.

[6] Basalay M. V., Yellon D. M., Davidson S. M. (2020). Targeting myocardial ischaemic injury in the absence of reperfusion. *Basic Research in Cardiology*.

[7] González-Montero J., Brito R., Gajardo A. I. J., Rodrigo R. (2018). Myocardial reperfusion injury and oxidative stress: therapeutic opportunities. *World Journal of Cardiology*.

[8] Bugger H., Pfeil K. (2020). Mitochondrial ROS in myocardial ischemia reperfusion and remodeling. *Biochimica et Biophysica Acta: Molecular Basis of Disease*.

[9] D’Oria R., Schipani R., Leonardini A. (2020). The role of oxidative stress in cardiac disease: from physiological response to injury factor. *Oxidative Medicine and Cellular Longevity*.

[10] Hausenloy D. J., Chilian W., Crea F. (2019). The coronary circulation in acute myocardial ischaemia/reperfusion injury: a target for cardioprotection. *Cardiovascular Research*.

[11] Nobian A., Mohamed A., Spyridopoulos I. (2019). The role of arginine vasopressin in myocardial infarction and reperfusion. *Kardiologia Polska*.

[12] Gori A. M., Cesari F., Marcucci R. (2009). The balance between pro- and anti-inflammatory cytokines is associated with platelet aggregability in acute coronary syndrome patients. *Atherosclerosis*.

[13] Karpov R. S., Popov S. V., Markov V. A. (2005). Autologous mononuclear bone marrow cells during reparative regeneratrion after acute myocardial infarction. *Bulletin of Experimental Biology and Medicine*.

[14] Frangogiannis N. G. (2022). Transforming growth factor-*β* in myocardial disease. *Nature Reviews Cardiology*.

[15] Huang S., Frangogiannis N. G. (2018). Anti-inflammatory therapies in myocardial infarction: failures, hopes and challenges. *British Journal of Pharmacology*.

[16] Blair H. A., Deeks E. D. (2017). Abatacept: a review in rheumatoid arthritis. *Drugs*.

[17] Cagnotto G., Willim M., Nilsson J.-Å. (2020). Abatacept in rheumatoid arthritis: survival on drug, clinical outcomes, and their predictors—data from a large national quality register. *Arthritis Research & Therapy*.

[18] Bozec A., Luo Y., Engdahl C., Figueiredo C., Bang H., Schett G. (2018). Abatacept blocks anti-citrullinated protein antibody and rheumatoid factor mediated cytokine production in human macrophages in IDO-dependent manner. *Arthritis Research & Therapy*.

[19] Fukue R., Okazaki Y., Gono T., Kuwana M. (2022). Abatacept downregulates Fc*γ* receptor I on circulating monocytes: a potential therapeutic mechanism in patients with rheumatoid arthritis. *Arthritis Research & Therapy*.

[20] Kim M. J., Lee S.-K., Oh S. (2022). Efficacy of Abatacept versus tumor necrosis factor inhibitors in anti-citrullinated protein antibody-positive patients with rheumatoid arthritis: results from a Korean Nationwide Biologics Registry. *Rheumatology and Therapy*.

[21] Verma V. K., Malik S., Mutneja E., Sahu A. K., Bhatia J., Arya D. S. (2020). Attenuation of ROS-mediated myocardial ischemia–reperfusion injury by morin via regulation of RISK/SAPK pathways. *Pharmacological Reports*.

[22] Ohkawa H., Ohishi N., Yagi K. (1979). Assay for lipid peroxides in animal tissues by thiobarbituric acid reaction. *Analytical Biochemistry*.

[23] Moron M., Depierre J., Mannervik B. (1979). Levels of glutathione, glutathione reductase and glutathione S-transferase activities in rat lung and liver. *Biochimica et Biophysica Acta (BBA) - General Subjects*.

[24] Marklund S., Marklund G. (1974). Involvement of the superoxide anion radical in the autoxidation of pyrogallol and a convenient assay for superoxide dismutase. *European Journal of Biochemistry*.

[25] Aebi H. (1984). Catalase in vitro. *Methods in Enzymology*.

[26] Suchal K., Malik S., Khan S. I. (2017). Protective effect of mangiferin on myocardial ischemia-reperfusion injury in streptozotocin-induced diabetic rats: role of AGE-RAGE/MAPK pathways. *Scientific Reports*.

[27] Mutneja E., Verma V. K., Malik S. (2020). Erdosteine salvages cardiac necrosis: novel effect through modulation of MAPK and Nrf-2/HO-1 pathway. *Journal of Biochemical and Molecular Toxicology*.

[28] Verma V. K., Malik S., Narayanan S. P. (2019). Role of MAPK/NF-*κ*B pathway in cardioprotective effect of Morin in isoproterenol induced myocardial injury in rats. *Molecular Biology Reports*.

[29] Mao S., Wang L., Ouyang W. (2016). Traditional Chinese medicine, Danlou tablets alleviate adverse left ventricular remodeling after myocardial infarction: results of a double-blind, randomized, placebo-controlled, pilot study. *BMC Complementary and Alternative Medicine*.

[30] Yin X., Yin X., Pan X. (2023). Post-myocardial infarction fibrosis: pathophysiology, examination, and intervention. *Frontiers in Pharmacology*.

[31] Kallikourdis M., Martini E., Carullo P. (2017). T cell costimulation blockade blunts pressure overload-induced heart failure. *Nature Communications*.

[32] Menezes-Rodrigues F. S., Errante P. R., Tavares J. G. P. (2019). Pharmacological modulation of b-adrenoceptors as a new cardioprotective strategy for therapy of myocardial dysfunction induced by ischemia and reperfusion. *Acta Cirurgica Brasileira*.

[33] Aydin S., Ugur K., Aydin S., Sahin I., Yardim M. (2019). Biomarkers in acute myocardial infarction: current perspectives. *Vascular Health and Risk Management*.

[34] Kesarwani P., Murali A. K., Al-Khami A. A., Mehrotra S. (2013). Redox regulation of T-cell function: from molecular mechanisms to significance in human health and disease. *Antioxidants & Redox Signaling*.

[35] Rufino A. T., Freitas M., Proença C., Ferreira de Oliveira J. M. P., Fernandes E., Ribeiro D. (2024). Rheumatoid arthritis molecular targets and their importance to flavonoid-based therapy. *Medicinal Research Reviews*.

[36] Romano E., Rosa I., Fioretto B. S., Matucci-Cerinic M., Manetti M. (2022). The role of pro-fibrotic myofibroblasts in systemic sclerosis: from origin to therapeutic targeting. *Current Molecular Medicine*.

[37] Li X., Yuan T., Chen D. (2018). Cardioprotective effects of puerarin-V on isoproterenol-induced myocardial infarction mice is associated with regulation of PPAR-*Υ*/NF-*κ*B pathway. *Molecules*.

[38] Zhang B., Wang H., Yang Z. (2020). Protective effect of alpha-pinene against isoproterenol-induced myocardial infarction through NF-*κ*B signaling pathway. *Human & Experimental Toxicology*.

[39] Mahmoud A. H., Taha N. M., Zakhary M., Tadros M. S. (2019). PTEN gene & TNF-alpha in acute myocardial infarction. *IJC Heart & Vasculature*.

[40] Tøllefsen I. M., Shetelig C., Seljeflot I., Eritsland J., Hoffmann P., Andersen G. (2021). High levels of interleukin-6 are associated with final infarct size and adverse clinical events in patients with STEMI. *Open Heart*.

[41] Alenazy M. F., Sharif-Askari F. S., Omair M. A. (2021). Abatacept enhances blood regulatory B cells of rheumatoid arthritis patients to a level that associates with disease remittance. *Scientific Reports*.

[42] Weisman M. H., Durez P., Hallegua D. (2006). Reduction of inflammatory biomarker response by Abatacept in treatment of rheumatoid arthritis. *The Journal of Rheumatology*.

[43] Garg S., Khan S. I., Malhotra R. K. (2021). Cardioprotective effects of azilsartan compared with that of telmisartan on an in vivo model of myocardial ischemia-reperfusion injury. *Journal of Biochemical and Molecular Toxicology*.

[44] Xiang M., Lu Y., Xin L. (2021). Role of oxidative stress in reperfusion following myocardial ischemia and its treatments. *Oxidative Medicine and Cellular Longevity*.

[45] Huang F., Song L. (2017). Nanoemulsion formulation of Abatacept for lupus nephritis therapy. *Tropical Journal of Pharmaceutical Research*.

[46] Ayza M. A., Zewdie K. A., Tesfaye B. A., Gebrekirstos S. T., Berhe D. F. (2020). Anti-diabetic effect of telmisartan through its partial PPAR*γ*-agonistic activity. *Diabetes, Metabolic Syndrome and Obesity: Targets and Therapy*.

[47] Devan A. R., Nair B., Kumar A. R., Nath L. R. (2022). An insight into the role of telmisartan as PPAR-*γ*/*α* dual activator in the management of nonalcoholic fatty liver disease. *Biotechnology and Applied Biochemistry*.

[48] Kishimoto M., Suenaga J., Takase H. (2019). Oxidative stress-responsive apoptosis inducing protein (ORAIP) plays a critical role in cerebral ischemia/reperfusion injury. *Scientific Reports*.

[49] Ghannam K., Martinez Gamboa L., Kedor C. (2020). Response to Abatacept is associated with the inhibition of proteasome *β*1i expression in T cells of patients with rheumatoid arthritis. *RMD Open*.

[50] Derambure C., Dzangue-Tchoupou G., D’Agostino M. A., Lequerré T., Vittecoq O., Kuwana M. (2020). Gene expression regulated by Abatacept associated with methotrexate and correlation with disease activity in rheumatoid arthritis. *PLOS ONE*.

[51] Chang X., Zhang K., Zhou R. (2016). Cardioprotective effects of salidroside on myocardial ischemia–reperfusion injury in coronary artery occlusion-induced rats and Langendorff-perfused rat hearts. *International Journal of Cardiology*.

[52] Chorianopoulos E., Heger T., Lutz M. (2010). FGF-inducible 14-kDa protein (Fn14) is regulated via the RhoA/ROCK kinase pathway in cardiomyocytes and mediates nuclear factor-kappaB activation by TWEAK. *Basic Research in Cardiology*.

[53] Naidu S., Vijayan V., Santoso S., Kietzmann T., Immenschuh S. (2009). Inhibition and genetic deficiency of p38 MAPK up-regulates heme oxygenase-1 gene expression via Nrf2. *The Journal of Immunology*.

[54] Zhou Z., Zhou B., Chen H., Lu K., Wang Y. (2021). Oxidative stress activates the Nrf2-mediated antioxidant response and P38 MAPK pathway: a possible apoptotic mechanism induced by BDE-47 in rainbow trout (*Oncorhynchus mykiss*) gonadal RTG-2 cells. *Environmental Pollution*.

[55] Khalifa A. A., El Sokkary N. H., Elblehi S. S., Diab M. A., Ali M. A. (2022). Potential cardioprotective effect of octreotide via NOXs mitigation, mitochondrial biogenesis and MAPK/Erk1/2/STAT3/NF-k*β* pathway attenuation in isoproterenol-induced myocardial infarction in rats. *European Journal of Pharmacology*.

[56] Iqubal A., Khan A., Laeeq A., Malhotra K., Ansari M. A., Haque S. E. (2021). Recent updates on current and upcoming biomarkers for cardiovascular diseases. *Current Pharmaceutical Design*.

