# Supplementary material for: Abatacept: A Promising Repurposed Solution for Myocardial Infarction-Induced Inflammation in Rat Models
Source: Oxid Med Cell Longev. 2024 Mar 13;2024:3534104. doi: 10.1155/2024/3534104 (PMC11219209; doi:10.1155/2024/3534104)
Supplement: Supplementary 1 — shows the detailed list of primary antibodies. [file 3534104.f1.docx]

**Supplementary Table 1:** List of primary antibodies used in the experiments

| **Primary Antibody** | **Molecular Weight** | **Company (Cat. No.)** |
| --- | --- | --- |
| β-actin | 45 kDa | Cell Signaling Technology (8457S) |
| GAPDH | 37 kDa | Cell Signaling Technology (5174S) |
| P38 | 40 kDa | Cell Signaling Technology (9212S) |
| p-P38 | 43 kDa | Cell Signaling Technology (4511S) |
| JNK | 46 kDa | Santa Cruz Biotechnology, Inc. (sc-572) |
| p-JNK | 46 kDa | Santa Cruz Biotechnology, Inc. (sc-6254) |
| Erk1/2 | 42/44 kDa | Cell Signaling Technology (9102S) |
| p-Erk1/2 | 42/44 kDa | Cell Signaling Technology (4370S) |
| Nrf-2 | 64 kDa | Abcam, California, USA (ab137550) |
| HO-1 | 34 kDa | Abcam, California, USA (ab189491) |
| Hsp27 | 27 kDa | Cell Signaling Technology (2442S) |
| Hsp70 | 70 kDa | Santa Cruz Biotechnology, Inc. (sc-1060) |
| NF-κB | 60 kDa | Abcam, California, USA (ab86299) |
| p-NF-κB | 60 kDa | Abcam, California, USA (ab28856) |
| TGF-β | 41 kDa | Cell Signaling Technology (3711S) |
| Akt | 56 kDa | Abcam, California, USA (ab179463) |
| p-Akt | 56 kDa | Abcam, California, USA (ab131443) |
| NOX-4 | 67 kDa | Santa Cruz Biotechnology, Inc. (sc-55142) |
| Cytochrome-C | 14 kDa | Cell Signaling Technology (11940S) |
| Bcl_2_ | 26 kDa | Abbkine (abm40041) |
| Caspase 3 | 35 kDa | Cell Signaling Technology (9665S) |
| Bax | 20 kDa | Cell Signaling Technology (2772S) |
