# Supplementary material for: Abatacept: A Promising Repurposed Solution for Myocardial Infarction-Induced Inflammation in Rat Models
Source: Oxid Med Cell Longev. 2024 Mar 13;2024:3534104. doi: 10.1155/2024/3534104 (PMC11219209; doi:10.1155/2024/3534104)
Supplement: Supplementary 2 — shows the details of ELISA kits used in the experiments. [file 3534104.f2.docx]

**Supplementary Table 2:** List of kits used in the experiments

| **Kit** | **Company Name** | **Catalog No.** |
| --- | --- | --- |
| CK-MB | Coral Clinical Systems, India | 1102070210 |
| LDH | Coral Clinical Systems, India | 1102160025 |
| IL-6 | CusaBio Technology LLC, USA | CSB-E11987r |
| TNF | CusaBio Technology LLC, USA | CSB-E04640r |
| TUNEL | Thermofisher Scientific, USA | A23210 |
